# Addressing Self-Control Cognitions in the Treatment of Trichotillomania: A Randomized Controlled Trial Comparing Cognitive Therapy to Behaviour Therapy

**DOI:** 10.1007/s10608-016-9754-4

**Published:** 2016-02-02

**Authors:** Ger P. J. Keijsers, Joyce Maas, Amras van Opdorp, Agnes van Minnen

**Affiliations:** Behavioural Science Institute, Radboud University Nijmegen, P.O. Box 9104, 6500 HE Nijmegen, The Netherlands; Department of Clinical Psychological Science, University Maastricht, Maastricht, The Netherlands; Centre of Anxiety Disorders Overwaal, Nijmegen, The Netherlands

**Keywords:** Trichotillomania, Cognitive therapy, Behaviour therapy, Randomized controlled trial

## Abstract

People with trichotillomania often have persistent negative beliefs about giving into one’s habit. Central in the present study was the hypothesis that the follow-up effects of cognitive therapy (CT), in which these negative beliefs are directly addressed, are better compared to the follow-up effects of behaviour therapy (BT). Fifty-six trichotillomania patients were randomly assigned to either six sessions CT or BT. Forty-eight completed their treatment. Follow-up measurements took place after a 3 months treatment-free period, and at 12 and 24 months. CT and BT both resulted in clear reductions of trichotillomania symptoms (severity, urge, inability to resist, and negative beliefs) immediately after treatment. There were no differences between the groups. Following the treatment-free period, there was a reoccurrence of symptoms. In contrast to our expectation, we failed to show that CT compared to BT resulted in lower relapse rates after the treatment-free period.

## Introduction

In several previous studies we investigated the effects of brief behaviour therapy (BT) for patients suffering from trichotillomania and from excoriation disorder (Keijsers et al. 2006b; Schuck et al. 2010; Van Minnen et al. 2003). During these studies we became aware that patients frequently and spontaneously reported all sorts of beliefs about giving way to their unwanted habit. Patients mentioned, for example, that they were unable to resist the urge to scratch at skin irregularities or that pulling hairs helped them to concentrate while learning for an exam. These beliefs about giving way to one’s habit appeared stable over time and typically were held for many years. These beliefs are not restricted to patients with trichotillomania or excoriation disorder but are also reported by healthy controls with non-pathological unwanted habits such as nail biting or snacking (Maas et al. 2015a). We investigated these ‘automatic self-control cognitions’ in regard to unwanted habits and our findings supported two types: one is the belief that giving into the habit is rewarding: It offers comfort, help, pleasure, or peace of mind. The other is the belief that the urge to give into the habit is uncontrollable and giving into the habit cannot be averted (Maas et al. 2015a).

Interestingly, these two types of automatic beliefs have a characteristic that is often present in automatic cognitions: they are false, but they have a tendency to become true when one believes them to be true. That is, in general, it is not that hard to refrain from performing an unwanted habit such as hair pulling or skin picking in a particular situation at a particular point in time. A certain amount of effort or alertness is needed. Refraining from the habit becomes harder, however, when one continues this alert state of mind over a couple of hours. Effort and alertness have to be sustained. In that case, the belief that giving in is rewarding or that refraining from the habit is impossible, is likely to negatively affect patients’ motivation to sustain the effort. In effect, these beliefs are going to be confirmed when one gives into the habit.

During our clinical trials with patients with trichotillomania and excoriation disorder, we observed that patients generally are able to recall these self-control cognitions when asked to describe recent giving-in situations in detail, even though they often appear unaware of these cognitions at first. The role of these automatic cognitions in trichotillomania and excoriation disorder remains unclear, however. It is unknown whether these cognitions serve as maintaining factors in trichotillomania or excoriation disorder, meaning that they are part of the automatic processes involved in the regular performance of unwanted habitual behaviour, or whether they operate as justifications afterwards when patients are asked to recall what went through their minds at the moment that they gave into their habit. What is clear, however, is that the occurrence of these cognitions is positively related to symptom severity of these patients (Maas et al. 2015a). Even if these cognitions are afterward justifications, we noticed that when confronted with them, patients often become struck by the inconsistency of believing on a rational level that they can learn to stop their unwanted habit and believing on an automatic, spontaneous level that they need the habit and cannot resist it.

Intrigued by the above observations in patients with trichotillomania or excoriation disorder, we became interested in the possible therapeutic effects of addressing automatic self-control cognitions with regard to unwanted habitual behaviour by means of cognitive therapy (CT). There also was another, more acute reason to explore new treatment possibilities for trichotillomania. The short-term treatment effects of BT (including habit reversal) generally are good and better than those of other treatments such as serotonin-based medication (Duke et al. 2010). The results regarding long-term effects of BT are less consistent, however. High relapse rates in successfully treated trichotillomania patients have frequently (but not always) been reported (Diefenbach et al. 2006; Keijsers et al. 2006b; Lerner et al. 1998; Mouton and Stanley 1996; Rogers et al. 2014).

There are several possible explanations why BT is effective in reducing trichotillomania symptoms in the short run but fails to sufficiently do so in the long run. The effects of BT for trichotillomania are commonly attributed to the use of treatment interventions which weaken (extinguish) stimulus–response associations (e.g., Keuthen et al. 1999; Mansueto et al. 1997). Indeed, step-by-step changing the stimulus environment or interrupting the stimulus–response chain, leads to reduced urge to pull one’s hair and to reduced hair pulling. However, as long as these interventions are not sufficiently established, they require effort and attention. Self-control experiments demonstrate that the ability to exert continued self-control is limited (e.g., Baumeister et al. 2000). The first possibility is that after an initial treatment phase in which motivated patients successfully apply the first steps in their treatment program, the treatment continues to be effortful because it takes a while before stimulus–response associations have been sufficiently weakened.

A second possibility is that stimulus–response associations are successfully weakened by behavioural interventions, but subsequent relapses result from renewal effects. Findings from renewal experiments suggest that ‘extinction’ is context sensitive. A once learned fear or appetitive reaction, which has successfully been abated with the help of behavioural interventions within a treatment context (e.g., treatment sessions, treatment goals, self-monitoring of hair pulling), suddenly returns when the treatment context is replaced by the original acquisition context (sitting sadly on the coach) once again. This can be the case when successful behavioural treatment is discontinued. Renewal effects have been demonstrated in animal (e.g., Bouton and Bolles 1979; Bouton and Peck 1989) and in human (e.g., Conklin and Tiffany 2002; Nelson et al. 2011; Vansteenwegen et al. 2005) studies, they have been found for fear extinction and for abated appetite reactions (Bouton and Peck 1989; Conklin and Tiffany 2002), and they have been associated with the occurrence of relapse in the treatments of alcoholics, smokers, and pathological gamblers, even when they were abstinent for months or years (Conklin and Tiffany 2002).

Based on all these considerations, we wondered whether CT might be better suited to produce long lasting treatment effects compared to BT. Self-control cognitions are common in trichotillomania patients and systematically challenging these self-control cognitions appears less effortful than applying self-control techniques. Also, CT is less prone to renewal effects because CT operates through a high-order, conceptual reevaluation of meanings rather than by extinguishing stimulus–response associations (Keijsers et al. 2006a). Further, there is the possibility that the large short-term effects of BT are also mediated by changed beliefs about the patients’ ability to control their hair pulling behaviour. Systematically targeting these beliefs by means of CT may produce longer lasting treatment outcomes.

The present study aimed to explore the short-term and long-term treatment effects of addressing automatic self-control cognitions in trichotillomania patients by means of CT. Although cognitive interventions have been added to enhance the effect of habit reversal or BT treatment programs in a number of previous studies (see Snorrason et al. 2015 for an overview), none of these studies was designed to investigate whether CT results in lower relapse rates than those for habit reversal or BT. In order to minimalize the effects of confounds in our study, we applied a ‘pure’ form of CT without self-monitoring of hair pulling, self-control instructions, or additional behavioral interventions. The control condition consisted of BT based on self-control procedures which had been successfully applied and tested in previous studies (Van Minnen et al. 2003; Keijsers et al. 2006b).The research questions were as follows: (1) Does pure CT lead to reductions of self-control cognitions and trichotillomania symptoms? (2) Are relapse rates after treatment discontinuation smaller in patients treated with pure CT than in patients treated with BT, and lastly, (3) does a higher occurrence of self-control cognitions after treatment predict relapse at follow-up measurements? We expected both CT and BT to lead to decreases in trichotillomania symptoms. After a 3 months treatment-free period, we expected CT to show smaller relapse rates as compared to BT. We expected that higher occurrence of self-control cognitions at post-treatment measurement predicts higher relapse rates after a 3 months treatment-free period and at long term follow-up measurements. In order to test these hypotheses, patients diagnosed with trichotillomania were randomly assigned to either a six sessions, manual-based, pure CT or to a six sessions, manual-based, pure BT, both followed by a treatment-free period of 3 months. To explore the effects of self-control cognitions on treatment outcomes in the long run, patients were followed up at 12 months and at 24 months.

## Method

### Participants

Participants were self-referrals or were referred to an outpatient academic clinic specialized in the treatment of body-focused repetitive behaviour disorders between 2005 and 2010. Criteria for inclusion were a current main diagnosis of trichotillomania according to DSM-IV (APA 1994) and being older than 15 years. Patients with organic brain disease, suicidal intent, or past or present psychosis were excluded. Patients with comorbid disorders were included but with the understanding that the present study and treatments were only directed at treating trichotillomania. Further, patients had to agree that random assignment to one of two treatments was part of the procedure, that each treatment comprised six sessions only, and that each treatment was followed by a treatment-free period of 3 months. Patients were informed that CT was a new treatment for trichotillomania with as yet unknown effects and that BT was an established treatment for trichotillomania with good short-term effects but a real possibility of relapse during or after treatment. They were informed that after the treatment-free period, additional sessions could be scheduled, whenever they thought it necessary or helpful.

**Fig. 1:**
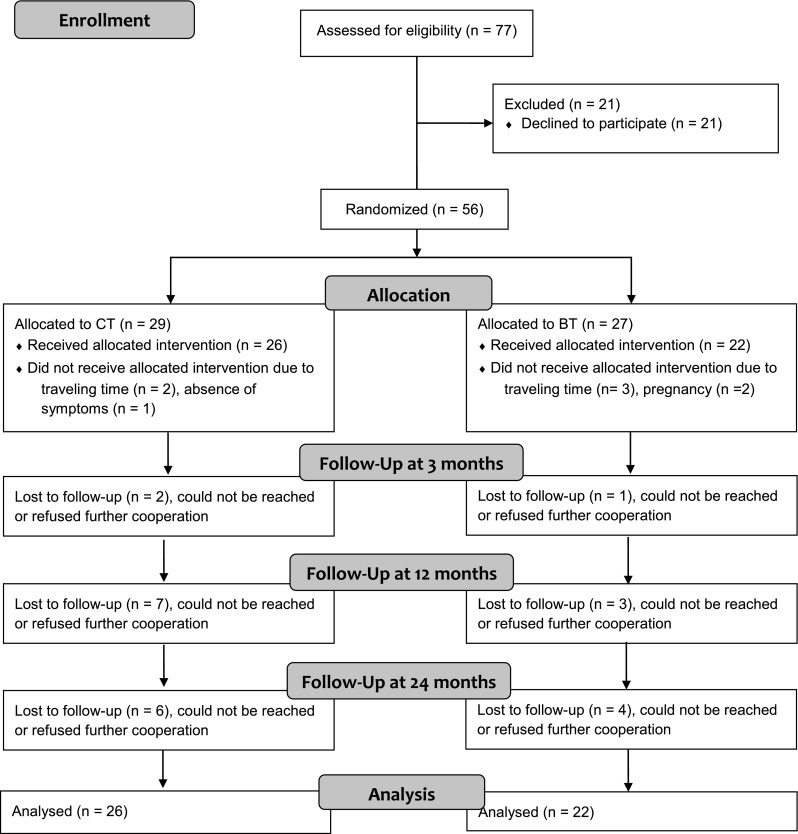
Flow of patients through each stage of the study

In total, 77 patients were selected for inclusion in the study. Twenty-one refused participation in the study and received treatment without additional measurements. Eight patients were treatment dropouts for several reasons (see Fig. 1). Considering the fact that we were interested in relapse after treatment over a longer period of time, we decided to focus on the 48 patients who had completed their treatment. Of these 48 patients, 18 (37.5 %) pulled hair from the scalp and 8 patients (16.7 %) from their eyebrows or lashes, or both. The other 22 patients (45.8 %) pulled hair from various parts of the body and in various combinations. Eighteen patients (37.5 %) reported to be consciously aware of their hair pulling most of the time, 24 (50.0 %) were sometimes aware and at other times unaware of their hair pulling, and the remaining six patients (12.5 %) were unaware of their habit most of the time. Forty-one patients (85.4 %) reported experiencing relief, pleasure, lust, or comfort during their hair pulling bouts and 36 patients (75.0 %) reported feeling guilty, ashamed, unpleasant, or angry after the hair pulling episode. These sample characteristics do not appear to differ from other trichotillomania samples reported elsewhere.

Of the 48 patients, 26 (54.2 %) were assigned to the CT condition and 22 (45.8 %) to the BT condition. Table 1 presents additional characteristics of the patients in both treatment conditions. Patients in CT and BT did not statistically differ with respect to sex, age, duration of trichotillomania symptoms, or number of additional DSM-IV disorders. Also, there were no statistically significant differences for education level, *χ*^2^(3) = 2.40, *p* = .48, number of dropouts, *χ*^2^(1) = 0.76, *p* = .38, or initial trichotillomania severity as measured with the Massachusetts General Hospital Hair pulling Scale, *t*(46) = −0.11, *p* = .92.

**Table 1:** Patients’ (completers) characteristics in cognitive therapy condition (CT) and behaviour therapy condition (BT), and tests for group differences

Characteristics	CT *N* = 26	BT *N* = 22	Total *N* = 48	Test statistics
Male (%)	2 (7.7)	1 (4.5)	3 (6.3)	*χ* ^2^ (1) = 0.20, *p* = .65
Age in years (*sd*)	32.6 (12.3)	30.4 (9.8)	31.6 (11.2)	*t* (46) = 0.46, *p* = .53
Symptom duration (*sd*)	19.9 (12.3)	17.7 (7.8)	18.9 (10.4)	*t* (46) = 0.71, *p* = .48
Additional mental disorder (%)^a^	5 (19.2)	7 (31.8)	12 (24.0)	*χ* ^2^ (1) = 0.46, *p* = .50
Anxiety disorder (%)	3 (11.5)	2 (9.1)	5 (10.4)	–
Mood disorder (%)	0	2 (9.1)	2 (4.2)	–
Somatoform disorder (%)	0	2 (9.1)	2 (4.2)	–
Other (%)	2 (7.7)	1 (4.5)	3 (2.2)	–

^a^According to DSM-IV criteria

Pre-and post-treatment outcomes were available for all patients, but regrettably not all follow-up data were complete. Of the initial 48 subjects, 3 failed to show up at the first follow-up assessment after 3 months. At 12 months follow-up evaluation, completed questionnaires could not be retrieved from ten patients. From another ten patients they could not be retrieved anymore at 24-months follow-up.

### Procedure

Patients considered eligible received a standardized clinical interview in which diagnostic criteria were verified (DSM-IV: APA 1994), clinical features established, and inclusion and exclusion criteria checked. Furthermore, they were offered oral and written information on the study and informed consent forms. If the patients decided to participate, the signed informed consent forms were collected during a second intake interview one week later. Informed consent was obtained from all participants included in the study. The Dutch version of the MINI-International Neuropsychiatric Interview (Sheehan et al. 1998) was completed and patients took part in a pre-treatment assessment, completing a number of self-report instruments (for details, see below). Subsequently, the patients were randomly assigned to either CT or BT. A block randomization was used with block sizes of ten without any covariates. Post-treatment assessments took place two weeks after the final treatment session, i.e., 14 weeks after the start of the treatment. Three months after the post-treatment assessment patients were invited for the first follow-up evaluation. After they had completed the self-report instruments, there was a meeting with one of the therapists in which progress was evaluated. Whenever patients or therapists considered it necessary, additional treatment sessions were offered. At this stage in the study, patients and therapists were free to use interventions from BT, CT or combinations of both. An average of 5.0 (*sd* = 3.5, range between 0 and 13 sessions) extra sessions were offered. The number of additional sessions did not statistically differ between CT and BT, *t* (42) = .38, *p* = .70). Twelve and 24 months after the post-treatment assessment, patients were requested by mail to again complete several self-report instruments. Post-treatment assessments and first follow-up evaluations were carried out by raters who were master-level students and fulfilled a clinical internship at the clinic. The raters were not blind to the treatment conditions but never rated their own patients.

### Treatments

Manual-based CT comprised six individual, 45-min treatment sessions held every other week. The treatment aimed to identify and challenge beliefs about one’s ability and motivation to exert control over one’s hair pulling. Socratic dialogue, behavioural experiments (carried out in vitro and not in vivo, to keep the treatment condition as pure ‘cognitive’ as possible) and other cognitive interventions were used to challenge beliefs that refraining from hair pulling is impossible or that hair pulling is helpful or otherwise sensible to do. Later on, patients were encouraged to formulate a motto (e.g., “I deserve beautiful hair”) to readily grasp and activate alternative beliefs. Towards the end of CT, beliefs regarding lapses in hair pulling were discussed in which patients were stimulated to challenge ‘snowballing’. ‘Snowballing’, a form of helpless thinking introduced by Baumeister et al. (1994), is associated with giving-up further attempts at self-control: (for example “See? I always knew I am unable to stop hair pulling. It might as well give up now”). Throughout the sessions, patients daily completed the automatic cognitions diary of Beck et al. (1979), which was adapted for trichotillomania. There were no instructions for self-monitoring of hair pulling and no self-control instructions or other behavioral interventions.

The control-condition was manual-based BT. Two previous trichotillomania studies applied this treatment and reported large effects immediately after treatment (Keijsers et al. 2006b; Van Minnen et al. 2003). BT also comprised six individual, 45-min treatment sessions held every other week and it aims at successful self-control. Through self-monitoring the patients learned to control unwanted behaviour in their own environment. The main components were stimulus control (organizing the environment), stimulus–response interventions (interrupting the response chain by other or incompatible activities, see also Azrin and Nunn 1973), and response consequences (self-rewards). Throughout the treatment the patient carried out a daily homework assignment that involved keeping record of the number of hairs pulled. The outcome of the assignment was discussed and graphically displayed during the sessions. Conscious awareness of hair pulling was increased by introducing aids such as band-aids around the fingers or tinkling bracelets worn around the wrists. Additionally, most patients were instructed to put on gloves in high-risk situations (stimulus control), which, besides increasing awareness, also prevented them from actual hair pulling. In the third session, the patient and therapist together selected stimulus–response interventions such as going for a walk, calling a friend or cleaning the kitchen. Furthermore, response consequences in the form of useful but tedious or unpleasant tasks (cleaning the bathroom, a 30-min jog) were jointly drawn up. In sessions 4 and 5 the stimulus–response interventions and response consequences were extended. In the final session, relapse prevention was addressed.

The treatments were delivered by therapists who were master-level students and worked to fulfil a clinical internship at the clinic. All therapists were carefully trained and delivered both treatments. They were weekly supervised by a licensed clinical psychologist/psychotherapist to ensure that they adhered to the manuals.

### Materials

Primary outcome measure was trichotillomania symptom severity, measured with Massachusetts General Hospital Hair pulling Scale (MGHHS: Keuthen et al. 1995). The MGHHS consists of seven items, rated for symptom severity from 0 to 4 and assesses several aspects of hair pulling during the previous 7 days: urge to pull, actual pulling, perceived control, and associated distress. The MGHHS (Keuthen et al. 2007) and its Dutch adaptation (Van Minnen et al. 2003) have good psychometric properties.

Urge to pull hair, ability to resist the urge, and occurrence of self-control cognitions were secondary outcome measures in the present study. To assess urge and ability to resist urge, two items of the Severity Urge Resistance Frequency questionnaire (SURF, based on Schuck et al. 2010) were used: SURF-urge consists of a ten centimetre Visual Analogue Scale (VAS-scale) with ‘not at all’ and ‘very strong’ printed at opposite sides. The item was phrased as follows: ‘How strong was the urge to pull hair in the last seven days?’ Respondents indicate their position on the line between the poles. The scores ranged from 0 (‘not at all’) to 100 (‘very strong’). SURF-resistance consisted of an identical VAS-scale with the same poles. The item now was phrased as follows: ‘How able were you in the last seven days to resist the urge to pull hair?’ The (pooled afterwards) scores, again, ranged from 0 (very strong) to 100 (not at all). The SURF is not yet validated. However, the items are face valid.

Occurrence of automatic ‘self-control cognitions’ was assessed with the Self-Control Cognitions Questionnaire (SCCQ: Maas et al. 2015a). The SCCQ comprises 11 items and two subscales, ‘Giving way is rewarding’ (SCCQ-rewarding, e.g., ‘After a hard day’s work, I often feel that I deserve to pull hair’ and ‘Resistance is impossible’ (SCCQ-impossible, e.g. ‘The urge to pull hair is so strong that I think I am not able to resist’). The SCCQ has good psychometric properties, it differentiates between pathological and non-pathological habits, and it is sensitive to treatment progress.

## Results

To analyse treatment effects immediately after treatment, a Repeated Measures MANOVA was conducted with Time (pre-treatment, post-treatment) as within-subject factor and Condition (CT, BT) as between subjects factor for the outcome variables MGHHS, SURF-urge, SURF-resistance, SCCQ-rewarding, and SCCQ-impossible. The effect for Time, *F*(5, 42) = 80.33, *p* < .0001, *η*^*2*^ = .90, was significant, the effects for Condition, *F*(5, 42) = 0.44, *p* = .81. *η*^*2*^ = .05, and for Time by Condition, *F*(5, 42) = 0.49, *p* = .78, *η*^*2*^ = .06, were not. Post-hoc analyses showed that Time effects were significant for all five outcome variables (all *p* values <.01 and all *η*^*2*^s ranging from .16 [SURF-urge] to .54 [SCCQ-impossible]), whereas the Condition effects (all *p* values were .34 or larger) and the Time by Condition effects (all *p* values were .23 or larger) were significant for none of the outcome measures. Means, standard deviations, and Cohen’s *d* for repeated measurements are reported in Table 2. Both treatments conditions resulted in a clear reduction of trichotillomania symptoms and a reduction of giving in cognitions. For none of the outcome variables there was a difference in treatment effects between the conditions.

**Table 2:** Means, standard deviations (sd), and effect sizes (Cohen’s d) of outcome measures in cognitive therapy condition (CT) and behaviour therapy condition (BT), measured before treatment (pre-treatment), after treatment (post-treatment), and at 3-months follow-up: N = 26 for CT and 22 for BT

Outcome measures		Pre-treatment	Post-treatment	3 months follow-up^a^	Cohen’s *d* pre − post	Cohen’s *d* pre − 3 months follow-up
MGHHS	CT (*sd*) BT (*sd*)	15.23 (4.36) 15.36 (4.38)	9.69 (6.75) 9.86 (6.56)	12.42 (6.25) 14.52 (6.19)	1.22 0.99	0.55 0.16
SURF-urge	CT (*sd*) BT (*sd*)	64.3 (29.2) 61.6 (22.2)	48.4 (30.4) 46.5 (30.6)	61.2 (32.1) 68.3 (27.1)	0.57 0.56	0.11 −0.26^b^
SURF-resistance	CT (*sd*) BT (*sd*)	59.8 (29.1) 57.6 (28.2)	41.6 (30.6) 39.1 (32.5)	48.0 (30.8) 60.9 (30.1)	0.61 0.61	0.39 −0.11^b^
SCCQ-rewarding	CT (*sd*) BT (*sd*)	8.35 (5.12) 7.95 (6.73)	4.23 (4.95) 4.18 (6.12)	5.41 (6.08) 5.33 (5.92)	1.12 0.91	0.66 0.74
SCCQ-impossible	CT (*sd*) BT (*sd*)	13.35 (4.97) 12.95 (3.86)	8.88 (5.94) 6.73 (5.55)	9.87 (5.61) 10.33 (5.17)	1.01 1.50	0.93 0.78

*MGHHS* Massachusetts General Hospital Hair pulling Scale, *SURF* Severity Urge Resistance Frequency Scale, *SCCQ* Self-Control Cognitions Questionnaire

^a^ n = 24 for CT and 21 for BT, ^b^ Strictly speaking, an effect size is not expressed as negative. Nevertheless, we chose to report it this way to emphasize an increase instead of a decrease of symptoms

To investigate whether relapse rates after the 3 months treatment-free period differed between the two treatment conditions, a second Repeated Measures MANOVA was conducted with Time (post-treatment, 3 months follow-up) as within-subjects factor. The effect for Time, *F*(5, 37) = 3.77, *p* = .007, *η*^*2*^ = .34, was significant in the opposite direction: Overall, there was a significant increase in symptoms. There were no significant effects for Condition, *F*(5, 37), = 0.38, *p* = .86, *η*^*2*^ = .05, or Time by Condition, *F*(5, 37) = 1.39, *p* = .25, *η*^*2*^ = .16. Post-hoc analyses showed that the time-effects were significant for all five variables (all *p* values <. 05 and *η*^*2*^s ranging from .15 [SCCQ-rewarding] to .28 [MGHHs]), whereas the Condition effects (all *p* values were .28 or larger) and Time by Condition effects (all *p* values were .32 or larger) were significant for none of the outcome measures. Thus, patients in both treatment conditions showed a relapse in trichotillomania symptoms and giving in cognitions. It should be noted, however, that the effect sizes for CT after the 3 months treatment-free period were larger than those found for BT in four of the five outcome measurements (see Table 2).

**Fig. 2:**
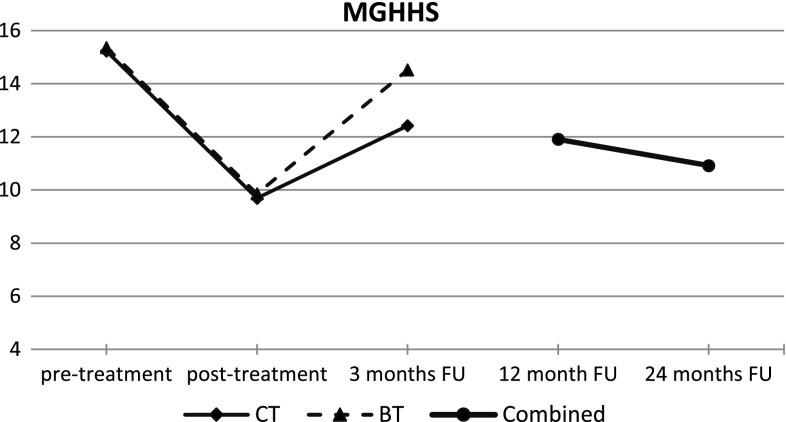
Means for Massachusetts General Hospital Hair pulling Scale (MGHHS) across all five measurements for cognitive therapy condition (CT) and behaviour therapy condition (BT) separately, and for both conditions combined

Figure 2 graphically displays the course of trichotillomania symptoms across all measurements including follow-up measurements after 12 and 24 months as measured with the MGHHS. With regard to the latter follow-up measurements, the findings for CT and BT are collapsed, since patients and therapists were free to use interventions from CT or BT in various combinations after the treatment-free period. Figure 2 shows a clear decrease of symptoms after treatment, a clear reoccurrence of symptoms at 3 months follow-up, and decreases of symptoms again at 12 months follow-up and at 24 months follow-up. Pre-post effect sizes (Cohen’s *d* for repeated measurements) for MGHHS were 0.68 at 12 months follow-up and 1.05 at 24 months follow-up. Similar patterns were found for SURF-urge, SURF-resistance, SCCQ-rewarding, and SCCQ-impossible. At 24 months follow-up, symptom levels were comparable to those found immediately after treatment.

In order to find out whether the occurrence of self-control cognitions immediately after treatment predicted treatment outcomes at the follow-up measurements, linear regression analyses were applied. SCCQ-impossible and SCCQ-rewarding scores, measured at the end of treatment, failed to significantly predict MGHHS-scores at 3 months follow-up, *F*(2, 41) = 0.84, *p* = .44, at 12 months follow-up, *F*(2, 33) = 0.13, *p* = .88, or at 24 months follow-up, *F*(2, 22) = 1.58, *p* = .23. The SCCQ sores also failed to predict SURF-urge (*p* values between .27 and .65) or SURF-resistance (*p* values between .20 and .85) follow-up scores. Also, there were no significant findings when Condition was added as an independent variable, or when regression analyses were carried out for CT and BT separately.

## Discussion

In the present study, we addressed automatic self-control cognitions in patients suffering from trichotillomania in a number of ways: We investigated the effects of a pure CT, aimed at changing patients beliefs about giving into hair pulling, we tested whether relapse rates in hair pulling were lower after CT than after a pure Behavior Therapy condition (BT), and we tested whether the higher presence/occurrence of self-control cognitions immediately after treatment predicted relapse rates found at follow-up evaluations. The first follow-up measurement took place after a period of 3 months in which there was no contact with the therapists, the latter ones were carried out 12 and 24 months after treatment discontinuation according to a naturalistic design, meaning that additional treatment sessions took place whenever patients and therapists agreed that they were needed.

Support for our hypotheses was mixed. CT as well as BT resulted in a clear reduction of trichotillomania severity (MGHHS), in reduced urge to pull hair (SURF-urge), in reduced inability to resist hair pulling (SURF-resistance), and in a reduction of automatic beliefs that one is unable to stop hair pulling (SCCQ-impossible) or that hair pulling is helpful or rewarding (SCCQ-rewarding) immediately after treatment. With respect to these findings, there were no differences between CT and BT. More importantly, and contrary to our expectations, relapse rates measured at 3 months follow-up were not smaller for CT than for BT. Further, higher occurrence of automatic self-control cognitions immediately after treatment failed to significantly predict higher relapse rates in any of the follow-up evaluations.

In order to explain these findings, it first has to be noted that six sessions of CT is as effective as six sessions of BT in reducing trichotillomania symptoms. This finding lends support for the assumption that beliefs about giving into one’s habit play a role in sustaining trichotillomania. Addressing them directly with CT without additional behavioural interventions, produces overall reductions of trichotillomania symptoms. This is the first study as far as we know, that demonstrates the effects of CT for trichotillomania without any added elements from BT. Interestingly, however, beliefs about hair pulling were also, and as strongly, affected by BT. BT also resulted in a significant reduction of automatic self-control cognitions. This might be understandable. When patients reduce their hair pulling with the use of BT techniques, they experience that resisting the urge to pull hair is possible and that the short-term rewards are tenuous at best compared to the long-term goals. Hence, successful BT also affects automatic beliefs about one’s habit and one’s ability for self-control.

A comparable picture emerges for the relapses after the 3 months treatment-free period. We expected that high relapse rates after successful treatment of trichotillomania were typical for BT. We reasoned that CT might be able to overcome a number of possible weaknesses of BT concerning the maintenance of treatment results: CT might be less effortful, might be less prone to renewal effects, and might have longer lasting effects due to addressing long held beliefs about giving into hair pulling. However, this was not the case. At the end of the treatment-free period, relapse rates after CT were as high as those after BT. Again, this might be understandable. When stimulus–response associations have not sufficiently been weakened, or when renewal effects occur, patients experience or re-experience urges to pull hair. The belief that they need to pull hair and are unable to refrain from it, are confirmed again and the earlier effects of CT become attenuated. Hence, successful CT is negatively affected by conditioned responses.

The conclusion that fits these findings best is that automatic beliefs about giving into hair pulling play a role in the sustenance of trichotillomania and interact with stimulus–response associations that have been learned over time. CT alone does not change the underlying mechanisms of trichotillomania strongly enough to result in smaller relapses as compared to BT. In addition, weak or strong beliefs about giving into hair pulling at the end of treatment and investigated in isolation, that is, without a combination with other maintaining factors, does not predict whether trichotillomania patients are able to maintain their treatment results on the short or long run. Despite our efforts to compare a pure CT with a pure BT, it is questionable to what extent both forms of treatment really tap into different mechanisms of change. This conclusion is in line with discussions regarding differential effects of CT and BT in the treatment of anxiety disorders. Based on a review of meta-analytic studies, Deacon and Abramowitz (2004) concluded that reliable conclusions on differential effects of CT and BT cannot be offered.

In regard to the practical implications of our findings, we regrettably have to conclude that we could not demonstrate that CT is able to overcome possible weaknesses of BT in regard to effect maintenance. It does not lead to smaller relapse rates. In line with the interpretation of our results, we would argue that CT and BT complement each other and that it is advisable to combine them, as has been done by others already. It should be noted however that also for the combination of BT and CT, no empirical data are available to support that a reduced number of relapses after treatment can be expected (Snorrason et al. 2015).

Several shortcomings of the present study have to be mentioned here. First, the patient sample was rather small, considering the fact that the effects of two well-established treatments were compared. Overall, pre to post effect sizes for CT were somewhat larger than for BT. We cannot tell whether meaningful differences between both treatment conditions would have emerged when we had been able to include more patients in the study. Second, SURF-urge and SURF-resistance, two of our secondary instruments to assess trichotillomania symptoms, have as yet not been properly validated. Third, extra efforts should have been made in the present study to control for treatment integrity of CT and BT, especially since the therapists delivered both treatments. The treatments were carried out according to detailed manuals and therapists were carefully trained to apply them. By means of supervision by an expert clinical psychologist once in 2 weeks, adherence to the manuals was ensured. For instance, therapists had to show the graphical display of monitored hairs of each patient in BT and they had to show completed diaries for automatic cognitions in CT. Nevertheless, treatment integrity was not checked and, hence, not confirmed by actual data. Forth, the present outcomes at the end of treatment were not as good as the outcomes reported in several previous clinical trials for trichotillomania and the relapse rates after 3 months follow-up were larger than previously found (e.g., Diefenbach et al. 2006; Keijsers et al. 2006b; Maas et al. 2015b; Woods et al. 2006). It is possible that the information we provided our patients with in the intake phase contributed to these findings. Please recall that in the intake phase we informed the patients that the effects of brief CT were unknown, that the effects of brief BT were better established but that relapses after BT were common. We told patients that CT and BT comprised six sessions and was followed by a 3 months treatment-free period after which additional sessions could be scheduled. In retrospect, we think it possible that this combination of information—mentioning relapse rates after treatment, inclusion of a strict treatment-free period, and promising additional treatment whenever necessary—may have triggered patients’ expectancy that the six sessions of treatment would either not suffice to reduce symptoms or would be a first step to reduce symptoms while additional sessions would be necessary to guarantee a full, long-term effect. This expectancy, in combination with the fact that we offered additional booster sessions after the 3 months treatment-free period may also account for the present findings that patients’ outcomes improved again at 12 months and 24 months follow-up measurements. In previous clinical trials in trichotillomania, follow-up findings generally show a decline of treatment effects over time, rather than a reduction of symptoms. The treatment effect at 24 months follow-up were comparable with those reported previously (Keijsers et al. 2006b). The addition of booster sessions likely contributed to further improvement on the long-term, but we cannot be sure, because neither in the present study, nor in other studies which successfully employed booster sessions, the effects of booster sessions were tested in a controlled way (e.g., Azrin et al. 1980; Rosenbaum and Ayllon 1981).

In sum, our study is the only study in trichotillomania in which the effects of pure CT have directly been compared with those of standard BT. Our findings showed that favourable treatment results can be achieved in patients suffering from trichotillomania by addressing automatic beliefs about giving into hair pulling with the use of cognitive interventions, but, regrettably, no extra possibility to reduce relapse rates after treatment could be detected. Our findings contribute to the view that immediate treatment effects in trichotillomania are good but stable maintenance of these effects over longer periods of time after treatment is uncertain. Further research is needed to address this issue. Planned booster sessions seem promising for further improvement on the long-term.
